# Mitotic activity: A systematic literature review of the assessment methodology and prognostic value in feline tumors

**DOI:** 10.1177/03009858241239566

**Published:** 2024-03-27

**Authors:** Christof A. Bertram, Taryn A. Donovan, Alexander Bartel

**Affiliations:** 1 University of Veterinary Medicine Vienna, Vienna, Austria; 2 Schwarzman Animal Medical Center, New York, NY; 3 Freie Universität Berlin, Berlin, Germany

**Keywords:** cat, methodology, mitosis, mitotic count, mitotic figure, mitotic index, neoplasia, outcome, prognosis, survival

## Abstract

Increased proliferation is a driver of tumorigenesis, and quantification of mitotic activity is a standard task for prognostication. This systematic review is an analysis of all available references on mitotic activity in feline tumors to provide an overview of the assessment methods and prognostic value. A systematic literature search in PubMed and Scopus and a nonsystematic search in Google Scholar were conducted. All articles on feline tumors that correlated mitotic activity with patient outcome were identified. Data analysis revealed that of the 42 eligible articles, mitotic count (MC, mitotic figures/tumor area) was evaluated in 39 studies, and mitotic index (MI, mitotic figures/tumor cells) in 3 studies. The risk of bias was considered high for most studies (26/42, 62%) based on small study populations, insufficient details of the MC/MI methods, and lack of statistical measures for diagnostic accuracy or effect on outcome. The MC/MI methods varied between studies. A significant association of MC with survival was determined in 20 of 28 (71%) studies (10 studies evaluated other outcome metrics or provided individual patient data), while 1 study found an inverse effect. Three tumor types had at least 4 studies, and a prognostic association with survival was found in 5 of 6 studies on mast cell tumors, 5 of 5 on mammary tumors, and 3 of 4 on soft-tissue sarcomas. MI was shown to correlate with survival for mammary tumors by 2 research groups; however, comparisons to MC were not conducted. Further studies with standardized mitotic activity methods and appropriate statistical analysis for discriminant ability of patient outcome are needed to infer the prognostic value of MC and MI.

Increased cell proliferation through self-sufficiency in growth and avoidance of growth-inhibitory signals is a key driver of tumorigenesis. Mitotic activity is a highly relevant measure for the growth fraction of tumor proliferation^56^ and is thus generally expected to correlate with a more aggressive biological behavior and less favorable patient outcomes for malignant tumor types. Quantification of mitotic figures (cells undergoing cell division visible in histological sections) is a standard task for tumor prognostication in veterinary pathology due to its high practicability and assumed high prognostic value.^16,33^ However, given the extensive availability of feline oncologic literature, identification of the recommended methods for measuring mitotic activity, as well as the prognostic relevance of these tumor parameters, can be difficult to ascertain for each individual tumor type.

There are 2 broad categories of measurement methods for mitotic activity, namely the mitotic count (MC) and the mitotic index (MI). While the MC represents the number of mitotic figures per tumor area, the proportion of tumor cells that have mitotic figures (among all tumor cells evaluated) is measured by the MI.^33,34^ Descriptions of the measuring methods of mitotic activity in oncologic research must be adequately detailed such that others can reliably and accurately reproduce the methods and data can be compared. Recent guidelines have defined key aspects of the MC, including the region of interest (ROI) within the tumor section, the size of the ROI in mm², the spatial arrangement of the fields of view within the ROI, and identification criteria of mitotic figures.^16,33^ Standardized methods for the MI have not been proposed for veterinary oncology. A summary of the methods applied in previous studies is needed to identify the degree of standardization. Diagnostic pathologists need to be aware of the different methods applied in the different tumor studies upon which they base their prognostic interpretation of the respective tumor type.

Regardless of the critical biological role of tumor cell proliferation in cancer development, the prognostic utility of mitotic activity in some feline tumors was not demonstrated.^29,50,62^ Potential explanations include the true lack of a prognostic relevance for mitotic activity in a particular tumor type and/or the use of nonstandardized, inconsistent, or inaccurate study methods, as well as nonrepresentative or small study populations. Validation studies are needed due to the high risk of bias (RoB) of observational studies and to validate the results in different study populations. Only then can final conclusions on the relevance of prognostic tests be drawn. Currently, there are no evidence-based recommendations for which tumor type the mitotic activity measurements should be routinely conducted as a solitary prognostic test.

This systematic review intends to fulfill the need for a scholarly overview of the methods and prognostic value of measuring mitotic activity in feline tumors. An extensive literature search was conducted to find all feline oncology studies that correlated the MC or MI with any type of patient outcome.

## Material and Methods

### Literature Search

**Figure 1.**
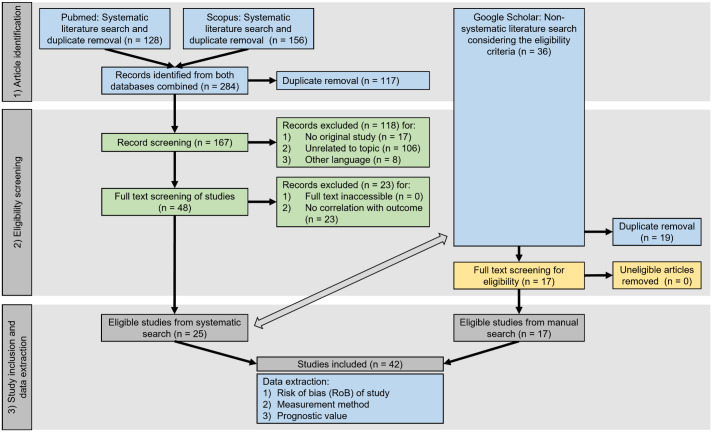
PRISMA flowchart^38^ summarizing the workflow of systematic (on the left) and nonsystematic (on the right) literature search, eligibility screening, and study inclusion and data extraction. The tasks in the blue boxes were conducted by the primary literature reviewer (CAB), the tasks in the green boxes were conducted by 2 literature reviewers in a blinded manner, and the tasks in the yellow boxes were conducted by the second literature reviewer (TAD). The double-sided arrow indicates the comparison of the articles identified through systematic and nonsystematic literature search necessary for removal of duplicates.

A literature search protocol was developed based on the Preferred Reporting Items for Systematic Reviews and Meta-Analyses (PRISMA) statement.^38^ The literature search consisted of (1) a systematic search in 2 databases (PubMed and Scopus) using predefined search terms (see the following sections) and (2) an nonsystematic search without a predetermined search strategy in a database (Google Scholar) to ensure literature saturation (Fig. 1). All identified references were screened for eligibility for study inclusion/exclusion by 2 authors (CAB and TAD) using the criteria as summarized in Table 1.

**Table 1.** Summary of the eligibility criteria for the two screening steps of the systematic literature search.

Screening Step	Decision Category		Inclusion Criteria	Exclusion Criteria
Title-abstract	(1) Study design		Original study, peer-reviewed	Case reports, reviews
	(2) Topic	(a) Species	Cat/feline	Other species
		(b) Tumor	Spontaneous tumors	Experimentally induced tumors
			Malignant tumors with potential for metastasis	Benign tumors
		(c) Prognostic test	Mitotic count (MC), mitotic index (MI)	No mitotic activity measurement
	(3) Language of main text		English or German	Other language
Full text	(1) Article accessibility		Article accessible	Article inaccessible
	(2) Topic	(a) Patient outcome	Correlation of the MC/MI with survival, tumor progression, metastasis, or recurrence	No correlation of the MC/MI with patient follow-up

For the systematic literature search, one author (CAB) searched PubMed (1950 to present) and Scopus (1970 to present) on June 9, 2022. The search string was built based on 2 topics for Who (animal) and What (prognostic test) resulting in the following search strings: (cat OR cats OR feline) AND (mitotic count OR mitotic index). The records identified in each database using these 6 combinations were exported to Endnote (Version X9.3.3) and sorted alphabetically based on their title to easily identify duplicates, which were subsequently removed. Two separate eligibility screening steps, the title-abstract and full-text screening, were done in the web application Rayyan^37^ by 2 literature reviewers in a blinded manner with the exception of one article that was written in German, which was only reviewed by CAB. Any disagreement between the two literature reviewers in the two screening steps were solved by joint full-text review and discussion. The artificial intelligence applications of Ryyan were not used for this study. Only articles that reported statistical results on the prognostic value of mitotic activity as a solitary parameter (ie, not in combination with other parameters, such as in a grading system) or provided individual patient data were included in this systematic review. Indirect assumptions on the prognostic value by correlation of the mitotic activity with other established prognostic parameters are not reliable and were not considered eligible.

The nonsystematic literature search was conducted from May to June 9, 2022. The database Google Scholar was searched without a predetermined search strategy and eligibility screening of only the first 100–200 of the search results (sorted by relevance). Search terms for Google Scholar were numerous and included “outcome,” “prognosis,” “survival,” and relevant tumor types such as “mast cell tumor,” “soft tissue sarcoma,” and “melanoma”. In addition, the citing references of the articles of interest (“cited by” search in Google Scholar) and articles cited in articles of interest for statements on the prognostic value of the MC were examined. Articles of potential interest were identified through the title and abstract, and the full text, if available, was screened for the same eligibility criteria as stated earlier. The records and full text of those articles that met the eligibility criteria were extracted, and duplicates from the systematic literature search were removed. Eligibility for inclusion of the remaining articles was verified by the second literature reviewer (TAD).

### Data Extraction and RoB

Data extraction of the relevant information from the articles that were included in the systematic review was performed by CAB. For each study, the citation information, year of publication, journal type (journal focusing on veterinary pathology, ie, *Veterinary Pathology*, *Journal of Comparative Pathology*, and *Journal of Veterinary Diagnostic Investigation*, versus other journals), and tumor type evaluated were recorded. Subsequently, information regarding the RoB, mitotic activity measurement method, and prognostic value (association of the MC/MI with patient follow-up) was extracted. Studies on MC and MI were analyzed separately.

RoB from each article was evaluated regarding the information on mitotic activity. Clear decision criteria, as listed in Supplemental Table S1 and summarized in the following parts of the article, were developed based on previous recommendations.^6,27,33,52,58^ These decision criteria were intended to be straightforward, applicable for this specific systematic review, and concede to the current practice of prognostic studies. We acknowledge that current recommendations for future studies might apply stricter criteria than we did for this RoB evaluation. This was considered necessary to enable rating of low RoB in at least some studies. We grouped the decision criteria into 4 domains that are critical when conducting a prognostic study: (1) study population, (2) outcome assessment, (3) mitotic activity measurement method, and (4) data analysis. The overall RoB was rated by combining all four domains. While a high RoB of domain 3 (although being highly important for replicability of the results) was considered less severe than the other domains, the combined score could only be one level higher than the lowest score.

Domain 1, the study population was mainly assessed based on the sample size for each outcome event but also on the selection bias of the patients and the availability of descriptions of patient characteristics. We based the threshold between high and moderate risk for the size of the study population on the recommendations for multivariate models (at least 10 cases of each event per variable included in the model)^58^ but reduced the threshold further (to 7) in order to accommodate the low case numbers available for most prognostic studies in veterinary medicine.

Outcome assessment (domain 2) was largely based on the method and duration of clinical patient follow-up, the similarity of treatment regimens, and the confirmation of the outcome event (such as cause of death). Questionnaires to submitters or outcome information extracted from medical records, representing the most common methods in previous prognostic studies, were considered to have a moderate RoB. Survival, tumor progression, or metastasis was considered a more appropriate outcome metric for mitotic activity than local tumor recurrence, which might be more associated with invasiveness of the tumor and local tumor control than with malignancy.

Mitotic activity measurement (domain 3) methods were evaluated based on completeness of the described methods and the assumed consistency of mitotic activity measurement. Whether the mitotic activity methods were consistent with recent recommendations^16,33,34^ was not considered in the evaluation, as these recommendations are not evidence-based. A complete MC method description, which allows good reproducibility of the approach, provides details on the evaluation location within the tumor section, the area evaluated (in mm² or equivalent), and the spatial arrangement of individual high-power fields (HPFs) that were evaluated.

Domain 4 (data analysis of the studies) was primarily based on the ability to interpret the discriminant ability of mitotic activity following the recommendations of a recent review on statistical analysis.^6^ The complete description of statistical methods applied and complete reporting of results for all available outcome metrics and methods for prognostic threshold determination were also considered.

## Results

### Study Selection

The literature selection process is summarized in Fig. 1. From 167 unique references found by the systematic database search, 25 articles remained eligible. Disagreement between the two literature reviewers only occurred for 1 article during the title-abstract screening steps. Based on a group discussion, this article was excluded as it evaluated a benign tumor without reported malignant biological behavior in the study. The nonsystematic literature search identified 17 additional articles for a total of 42 articles included in the review.^2,7,8,10–15,18,20,22–25,28–31,35,36,39–51,53,55,57,59–63^

### Study Characterizations

All articles were written in English except for one article, which was written in German.^25^ Sixteen of the 42 (38%) articles were published in journals with a focus on veterinary pathology (*Veterinary Pathology*, *N* = 13; *Journal of Comparative Pathology*, *N* = 1; *Journal of Veterinary Diagnostic Investigation*, *N* = 1), and 26 of 42 articles (62%) were published in other journals.

**Figure 2.**
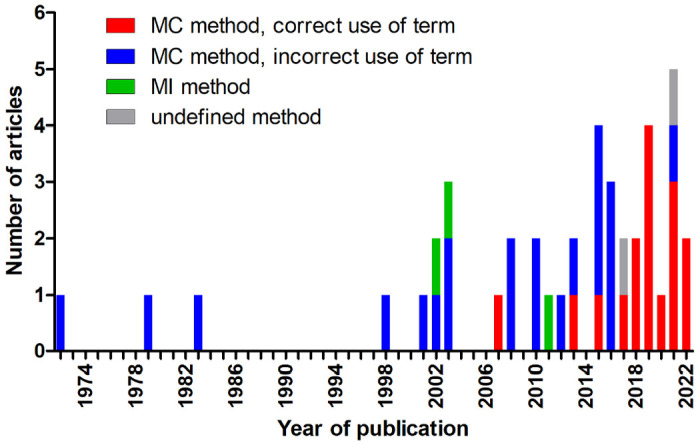
Stacked bar chart of the number of publications on the mitotic count (MC) and mitotic index (MI) included in this systematic review separated by year of publication. Any term other than MC was considered incorrect for measuring the number of mitotic figures per unit area. The year 2022 only includes January to June 9th.

The mitotic activity measurement methods were described in 40 of 42 articles (95%), and the measuring method was not specified in 2 articles (5%).^29,30^ According to our definition, the MC, that is, the number of mitotic figures per tumor area, was described in 37 articles.^2,7,8,10–15,18,20,22–25,28,31,35,36,39,40,42–51,53,59–63^ We assume that the two articles without specified methods^29,30^ also conducted the MC, as the data were taken retrospectively from medical records (total: 39/42 articles; 93%). Three^41,51,55^ (7%) studies measured the MI, that is, the percentage of mitotic figures per number of tumor cells. The number of publications on mitotic activity increased notably over the last decade (Fig. 2).

While the three studies on the MI used the correct terminology, the 39 studies which employed the MC used various and sometimes multiple terms, including MC (*n* = 16, 41%), MI (*n* = 16, 41%), number of mitoses (*n* = 4, 10%), mitotic rate (*n* = 3, 8%), mitoses (*n* = 2, 5%), and mitotic activity (*n* = 1, 3%). In January 2016, a guest editorial in *Veterinary Pathology*^34^ pointed out the proper use of the terminology MC and MI. While only 13% (3/23) of the articles published before 2017 used the correct term “MC,” 81% (13/16) published after 2016 used the correct term. Incorrect use of terminology after 2016 occurred only in non–pathology-focused journals.

## Mitotic Count

The 39 articles on the MC investigated a variety of different tumor types or tumor groups (Supplemental Table S2–S5). Tumor types/groups with multiple studies included mast cell tumors (*N* = 9; cutaneous, *N* = 7, pleomorphic cutaneous, *N* = 1; intestinal, *N* = 1), soft-tissue sarcomas (*N* = 6; injection-site sarcoma, *N* = 3; cutaneous, *N* = 1; piloleiomyosarcoma, *N* = 1; fibrosarcoma of mostly skin but also of oral cavity and bone, *N* = 1), malignant mammary tumors (*n* = 5), and melanocytic tumors (*N* = 5; nonocular, *N* = 3; nasal planum, *N* = 1; iris, *N* = 1).

### RoB of the Studies on MC

The RoB for all studies is summarized in Table 2, and the RoB for each article is listed in Supplemental Table S2. The bias of the study population was greatly influenced by small numbers of cases per outcome group (*N* < 7), which was particularly relevant for tumor types with a single study (low: 0, moderate: 6, high: 10), as compared to tumor types with multiple studies (low: 7, moderate: 11, high: 5). Outcome information was mostly obtained through questionnaires or medical records. Only 1 study conducted a postmortem examination after the death of the patient,^7^ and 2 studies conducted prospective clinical follow-up or questionnaires at regular intervals.^7,62^ MC methods were often incompletely described, as detailed in the next section, leading to a high RoB in 24 of 39 articles (62%). Data analysis (correlation of the MC with outcome) was often restricted to the *P* value approach (mostly log-rank test), which does not allow evaluation of the discriminant ability of the test.^6^ Of 29 studies that statistically correlated the MC with survival, 11 (38%) provided only results of tests of significance (*P* value approach), and results of other statistical tests that measure prognostic accuracy (such as cox regression, or area under the receiver operator characteristic curve [AUC]) or graphical illustrations (such as Kaplan-Meier curves or scatterplots) were not available. Of these 11 articles, 4 (36%) reported the *P* value, and 7 (64%) stated only that the *P* values were above the level of significance (“*P* > .05” or “not significant”) without providing the actual *P* value.

**Table 2.** Summary of the risk of bias (RoB) for each evaluated domain (D1–4) and overall RoB for all studies on the mitotic count (MC) in feline tumors combined (*N* = 39).

RoB	Number and Percent of Articles				
	D1: Study Population	D2: Outcome Assessment	D3: MC Method	D4: Data Analysis	Overall (D1–4)
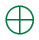 Low	7 (18%)	3 (8%)	6 (15%)	2 (5%)	1 (3%)
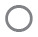 Moderate	17 (44%)	28 (72%)	9 (23%)	15 (38%)	12 (31%)
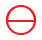 High	15 (38%)	8 (21%)	24 (62%)	22 (56%)	26 (67%)

### MC Methods

The MC values were taken from medical records (pathology reports) in 3 of 39 (8%) studies.^8,29,30^ The remaining 36 (92%) studies determined the MC values based on their study protocol using hematoxylin and eosin stain or, in one study, hematoxylin, eosin, and saffron stain.^12^ Bleaching of melanin pigment was performed for melanocytic tumors if required.^39,42,49^ In 5 studies,^35,39,40,49,57^ histopathological evaluation of the MC was done by multiple (2–4) pathologists with consensus in discordant cases (*N* = 2), simultaneous evaluation (*N* = 1), calculation of the mean value (*N* = 1), or unknown details of consensus for one study. None of the studies reported the use of digital microscopy or automated image analysis.

**Figure 3.**
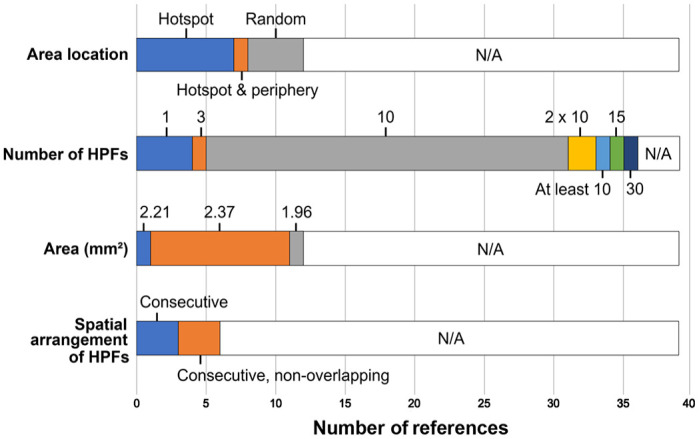
Stacked bar chart of the mitotic count methods used in 39 eligible studies on feline tumors regarding the location of the evaluated field(s) within the tumor section, number of high-power fields (HPFs) enumerated, area in mm² (based on the field number of the light microscope) enumerated, and spatial arrangement of the individual HPFs. N/A, not available.

The specific MC methods are summarized in Fig. 3 and detailed for each study in Supplemental Table S3. Generally, a somewhat higher proportion of articles published after 2016 and in pathology-focused journals described the individual aspects of the MC methods than those published before 2017 and in non–pathology-focused journals (see summary text to the Supplemental Table S3).

### Prognostic Value of the MC

The 39 references included outcome information by providing statistical results in the article (*N* = 29), tables with individual patient data (*N* = 6), or both (*N* = 4). Information on survival time (overall or tumor-specific survival) was available in 32 articles (28 studies with statistical analysis and 4 studies with individual patient data, Supplemental Tables S4 and S6), tumor progression (metastasis and/or recurrence) in 8 articles, metastasis in 6 articles, and recurrence in 8 articles (Supplemental Tables S5 and S6). The evaluated number of cases with follow-up per study ranged from 4 to 342 (mean: 44; median: 30).

Prognostic cutoff values for the MC were provided in 23 studies (59%), whereas only 12 (52%) indicated how this classification was created: median of the MC values (*N* = 4),^35,47,48,62^ mean of the MC values (*N* = 1),^44^ tertiles of the MC values (*N* = 1),^25^ receiver operating characteristic curve (*N* = 4),^12,14,40,45^ based on a previous study (*N* = 1),^18^ or “based on data analysis and literature” (*N* = 1).^49^

**Figure 4.**
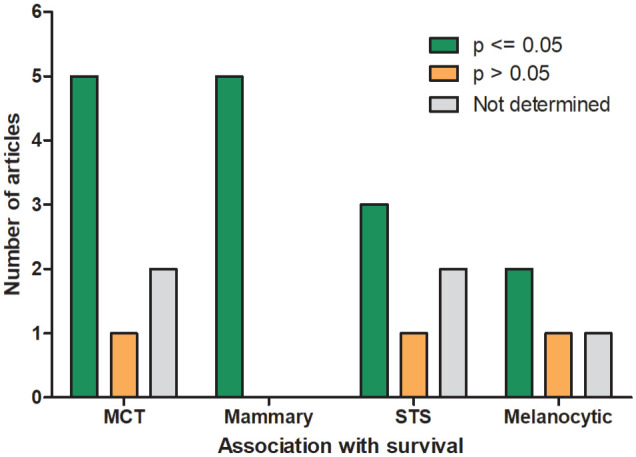
Prognostic significance of the mitotic count for survival (*P* value approach) for the feline tumor types with more than 3 articles. Study results with a *P* value of ≤.05 are considered significant. MCT, cutaneous mast cell tumors; mammary, malignant mammary tumors; STS, (sub)cutaneous soft-tissue sarcomas (one study included a few noncutaneous sarcomas); melanocytic, nonocular melanocytic tumors.

Regarding survival, 20 of 28 (71%) found a significant association (*P* value approach) of higher MCs with shorter survival, and 8 of 28 (29%) did not (Supplemental Table S6); 4 additional studies only provided individual patient data with the MC value and survival time of each patient. The study by Hammer et al^22^ found an inverse effect in salivary gland tumors, with higher MC values being significantly associated with longer survival. There were 4 tumor types with at least 3 studies evaluating survival. Higher MCs were associated with a shorter survival time or lower survival rates in 5 of 6 studies on mast cell tumors of the skin, 5 of 5 studies on mammary tumors, 3 of 4 studies on (sub)cutaneous soft-tissue sarcoma (one study included a few cases from noncutaneous locations), and 2 of 3 studies on nonocular melanocytic tumors (Fig. 4). The AUC was reported for 2 studies on mast cell tumors and suggested a high discriminant ability (AUC = 0.79 and 0.92).^14,45^

Tumor progression was significantly correlated with higher MCs in 4 of 6 studies (67%, Supplemental Table S6). A prognostic significance of the MC regarding occurrence of metastasis was found in 2 of 3 studies (67%, Supplemental Table S6). Tumor recurrence was significantly associated with higher MCs in 4 of 8 studies (50%, Supplemental Table S6). While recurrence was often evaluated for soft-tissue sarcomas (*N* = 5), only 2 of 5 (40%, Supplemental Table S6) found a prognostic significance for the MC.

### MI: RoB, Methods, Prognostic Value

The 3 articles on the MI examined feline mammary carcinoma.^41,51,55^ The overall RoB was judged to be moderate for all 3 studies (Supplemental Table S7).

Two studies were conducted by the same research group who used the same MI methods and presumably the same study population.^41,51^ These two studies used toluidine blue stain for enhancement of mitotic figures (as suggested by a previous study).^9^ For the MI (mitotic figures per 100 tumor cells), 10 HPFs at 250× magnification were selected in a hotspot location, and presumably photomicrographs were taken. Utilizing an image cytometry software, the total nuclear area and mean nuclear area of tumor cells were determined, the quotient of which was used to estimate the number of tumor cells.

The third study determined the MI from 1000 tumor cells in 8–10 HPFs selected at the periphery and hotspot tumor location.^55^ The MI was probably determined in Ki67-immunolabeled sections.

The three studies determined a significantly shorter tumor-specific survival time for cases with higher MI (Supplemental Table S8). All studies used the median MI values as the prognostic cutoff value for their study populations.

## Discussion

This systematic review analyzes measurement methods and prognostic value of the MC and MI in feline tumors. The evaluated studies found a significant association of poorer outcome with higher MCs in 50%–71% (depending on the outcome metric) instances and with higher MIs in all three instances. Overall, this suggests a prognostic relevance of mitotic activity in feline tumors. However, the ability to derive conclusions was limited. A general finding of the systematic review is that there is a paucity of literature for most tumor types, there are various mitotic activity measurement methods, and many studies lack relevant information to interpret the prognostic value of mitotic activity. As observational studies have bias in the study populations (case selection and outcome assessment) and as there is high rater variability in enumerating mitotic figures,^3,4,16^ multiple studies are crucial to confirm, modify, or reject research findings.^32,54^ There is a marked increase in the number of articles on the prognostic relevance of the MC over the last decade, and our tumor type–specific conclusions may be updated when more validation studies are available. Adherence of future prognostic studies to recommendations for conducting prognostic studies and standardized MC methods will facilitate systematic reviews and allow for a meta-analysis.

For this systematic review, a new RoB evaluation system was developed that is based on the critical aspects (domains) of conducting a prognostic study. Previous recommendations for prognostic studies were adapted to evaluate the MC/MI as a solidary prognostic test. We emphasize that the applied decision criteria might need to be adapted for future systematic reviews. One criterion that is particularly debatable is the size of the study population needed for sufficient evidence/confidence in the accuracy of the results. The appropriate case number used for a study may vary from our RoB criteria depending on, among others, the incidence of the tumor, biological behavior (proportion of cases with and without the outcome event), and the intended analysis (for example univariate vs. multivariate analysis). However, a low case number per outcome event will indicate a higher RoB regarding the representativeness of the study population, and caution should be taken when interpreting these data and applying these results to a patient. The results of any prognostic study, particularly those which are based on low case numbers, need to be validated. Combining the results of several studies on the same topic (similar to this systematic review) will increase the confidence in the conclusions, as long as the results are similar and the studies have different sources of bias. This means that availability of several studies, each with a small study population (ie, high RoB), might improve interpretability of the prognostic relevance of the test. We highlight that validation was lacking for most tumor types evaluated in this systematic review; thus, we do not provide interpretation of the prognostic relevance of mitotic activity for these tumor types. Particularly studies with small case numbers should provide individual patient data which allows a combined statistical analysis of multiple studies.

Not surprisingly, most studies evaluated the MC, which is much more practical than the MI in a diagnostic setting. However, the terminology for this measurement method has been inconsistently used in previous studies. Terms such as MI, mitotic rate, or mitoses should be avoided to denote enumeration of mitotic figures in a certain tumor area. The MI indicates that the mitotic density was determined relative to the tumor cell density.^34^ Rate is defined as the number of events over time (such as the heart rate: beats per 1 minute). Mitosis is the process of cell division, which cannot be seen under the light microscope. The morphology of dividing cells within the M phase of the cell cycle is visible to pathologists as structures called “mitotic figures.”

The MC methods were quite variable, and relevant information was often lacking. Since 2016, some commentaries and guidelines have been published^16,33,34^ to increase awareness of critical methodological aspects of the MC needed for standardization. Particularly, the appropriate measure for the area evaluated (mm² instead of HPFs) was highlighted. Of note, recent articles provide information concerning area more often, showing that researchers are increasingly aware of the need to provide the details of the MC methods. A recent guideline document, developed under the auspices of the Veterinary Cancer Guidelines and Protocols (VCGP) group,^33^ provides standard recommendations for each of the critical methodological aspects of the MC (hotspot location; consecutive, nonoverlapping fields of view; 2.37 mm² area), which were the most common methods used in the feline studies with provided information. However, other methods were not uncommon, and it is still largely unknown which method is best from a prognostic standpoint and with regard to reproducibility. While the best method is still debatable, we highlight that studies need to report the precise methods on all aspects of the MC: location within tumor, spatial arrangement of the field of view, and area in mm².

A prognostic value of the MC for survival could only be evaluated for a few tumor types/groups with multiple studies. Using the *P* value approach, a significant association with survival was found by all or most studies on cutaneous mast cell tumors, mammary tumors, and soft-tissue sarcomas. For all the other tumor types/groups, the prognostic value of the MC remains unproven considering the lack of validation. A difficulty regarding the comparison/combination of studies is that many studies evaluated a heterogeneous group of tumors (such as case inclusion of fibrosarcoma of skin, bone, and the oral cavity^7^), while other studies evaluated specific tumor entities (such as cutaneous piloleiomyosarcoma^20^). Combined evaluation of studies on the different tumor subtypes and/or locations was deemed necessary in order to create tumor groups with multiple studies that could be compared. For this reason, we have provided all the extracted information in supplemental tables.

Interpretation of the prognostic value based on the *P* value approach (hypothesis testing by comparing the average mitotic activity measurements of two outcome groups) has considerable limitations as it depends on the case numbers of the study population and event rate and does not allow evaluation of the prognostic accuracy/discriminant ability of the MC.^6,19^ Considering the case numbers relative to the *P* values, the discriminant ability of the MC of mammary tumors might be somewhat less than that for other tumor types; however, this needs to be verified by statistical tests of prognostic accuracy, such as the AUC value. Another limitation in interpreting the significance of the *P* value approach was that many studies only expressed a statement of inequality (*P* > .05 or “not significant”) and did not provide the actual *P* value, which can range between.05 and 1.0.^19^ *P* values above .05 do not rule out a relevant prognostic accuracy, particularly if the study population and/or the event rate was small. Statistical analyses that better describe the discriminant ability (such as hazard ratios and AUC, sensitivity and specificity values)^6^ and/or graphical illustrations (such as the Kaplan-Meier curve, scatterplots or receiver operating characteristic curves) were inconsistently reported, which precludes in-depth evaluation of the prognostic relevance of the MC. For future publications, authors are highly encouraged to use these tests of discriminant ability, particularly the AUC values, that facilitate interpretation of their results and comparison with other studies.

The MI was reported in only 3 studies on feline mammary tumors,^41,51,55^ 2 of which presumably used the same study population.^41,51^ While these two research groups suggest a prognostic value for mammary tumors, further studies are needed that provide statistical tests for discriminant ability in mammary tumors and evaluate further tumor types, such as mast cell tumors. Mammary tumors seem to be good candidates for the MI due to the variable tumor cell density resulting from cystic spaces, sclerosis, and necrotic areas. Adjustment by the tumor cell density may better reflect the mitotic activity of the tumor and may provide a more accurate prognosis; however, a prognostic benefit compared to routine MC has not been shown for feline mammary tumors. An alternative solution to adjust for variable tumor cell density is volume-corrected MCs,^21^ which has, however, not been evaluated as a prognostic test for tumors in veterinary medicine thus far.

The limitation of the MI is the additional time investment for counting the number of tumor cells, which hampers application in a routine diagnostic workflow. We consider automated image analysis, when it becomes widely available, very promising for facilitating evaluation of MI in the future. An increasing number of laboratories use digital microscopy for their diagnostic workflow,^5^ and development of deep learning-based image analysis algorithms, including those that can segment and count tumor cell nuclei,^17,26^ is of great research interest. Automated enumeration of tumor cell nuclei, possibly in combination with algorithmic detection of mitotic figures,^1,3^ would allow routine application of the MI.

## Conclusion

Mitotic activity is often considered one of the most useful histological prognostic tests for tumors. In cats, however, there is a paucity of literature to support this argument, and of the literature that exists, the argument is confounded by considerable RoB, high variability of the measurement methods of mitotic activity assessment, and restriction of statistical analysis to hypothesis testing (*P* values). More than two-thirds of the studies found prognostic significance of the MC for patient survival, indicating general relevance of this test. However, sufficient evidence of the prognostic value exists for few tumor types (cutaneous mast cell tumors, mammary tumors, and possibly soft-tissue sarcomas). For other tumor types, validation studies are lacking. Researchers should be encouraged to validate and publish findings in independent study populations and with appropriate statistical tests for prognostic accuracy (particularly the AUC analysis). Thus far, the MI has only been evaluated for feline mammary carcinoma, while a prognostic comparison to MC is lacking.

## Supplemental Material

sj-pdf-1-vet-10.1177_03009858241239566 – Supplemental material for Mitotic activity: A systematic literature review of the assessment methodology and prognostic value in feline tumors Supplemental material, sj-pdf-1-vet-10.1177_03009858241239566 for Mitotic activity: A systematic literature review of the assessment methodology and prognostic value in feline tumors by Christof A. Bertram, Taryn A. Donovan and Alexander Bartel in Veterinary Pathology
