# Supplementary material for: Mitotic activity: A systematic literature review of the assessment methodology and prognostic value in feline tumors
Source: Vet Pathol. 2024 Mar 27;61(5):743–51. doi: 10.1177/03009858241239566 (PMC11370206; doi:10.1177/03009858241239566)
Supplement: sj-pdf-1-vet-10.1177_03009858241239566 – Supplemental material for Mitotic activity: A systematic literature review of the assessment methodology and prognostic value in feline tumors [file sj-pdf-1-vet-10.1177_03009858241239566.pdf]

Supplemental Materials

Mitotic activity: a systematic literature review of the assessment methodology and prognostic value in feline tumors

Christof A. Bertram, Taryn A. Donovan, Alexander Bartel

Supplemental Table S1. Criteria applied for the risk of bias (RoB) evaluation.

Quality criteria	RoB evaluation			
	⊕ Low	◯ Moderate	⊖ High	⊖ unknown
**Study population** (domain 1):	Cumulative evaluation of each criterion within domain 1			
Sample size (smallest event / total)	At least 15 cases per outcome event	Between 7 – 14 cases per outcome event	Less than 7 cases per outcome event	Number of cases per outcome event not provided
Inclusion and exclusion criteria (case definition)	Criteria clearly stated and low bias suspected (representative study population)	Criteria clearly stated and moderate bias suspected	Criteria clearly stated and high bias suspected	Criteria not stated
Patient characteristics and tumor (sub)types	Clearly described	Partially described	–	No information
**Outcome assessment** (domain 2):	Cumulative evaluation of each criterion within domain 2			
Outcome metrics for prognosis	Appropriate outcome metrics and definitions	Appropriate outcome metrics, insufficient definitions	Inappropriate outcome metric (only tumor recurrence)	–
Follow-up method (survival, disease progression)	Regular clinical follow-up until death or end of follow-up<br><br>Confirmation of cause of death by postmortem examination	Outcome extracted from medical records<br><br>Questionnaires to the submitting vet / owner	–	Method not stated
Confirmation of recurrence and metastasis	Confirmation by cytology or histology	Suspected metastasis based on diagnostic imaging<br><br>Suspected recurrence based on recurring mass in surgical location	Based on survey with unknown method of confirmation	Method not reported
Follow-up period and proportion lost to follow-up	Appropriate period for the tumor type and few cases lost to follow up	Short period or many cases lost to follow-up	Period considered too short for the tumor type and many cases lost to follow-up<br><br>No follow up (e.g. presence of metastasis at time of tumor removal)	Follo-up period and proportion lost to follow up not stated
Therapeutic treatment regimes	Only one treatment regimen for all patients; only surgical treatment is preferred	Different treatments, but similar between outcome groups	Different treatments between groups	Treatment not specified
**Mitotic activity method** (domain 3):	Cumulative evaluation of each criterion within domain 3			
Description of **mitotic count** methods (not applicable for studies on the mitotic index)	Information on area size (mm²) as well as area location, (and spatial arrangement) provided	Information of area size (in mm²) provided<br>Or<br>Information of area location and spatial arrangement	Only the number of HPF specified	No information provided
Description of **mitotic index** methods (not applicable for studies on the mitotic count)	Area selection (location, spatial arrangement) clear and number of cells or area size enumerated provided	Information on number of cells or area size enumerated provided	–	–

Consistency of MC / MI methods	New measurements determined for the study using predefined methods<br><br>All cases assessed by the same pathologist(s)	–	Mitotic counts taken from histopathology reports (with likely variable methods between pathologists)<br><br>Cases assessed by different pathologists	–
**Data analysis** (domain 4):	Cumulative evaluation of each criterion within domain 4			
Description of statistical methods	Clear description	Lack of some information	Lack of relevant information	No description
Outcome metrics tested	Results of statistical tests for prognostic value of MC / MI reported for all outcome metrics available in the study	Results reported for most outcome metrics available in the study	Results only reported for one of many outcome metrics available in the study (suspicious for selective reporting)	–
Relevant statistical tests (discriminant ability) performed, and results reported?	ROC curve and area under the curve, event rate, sensitivity and specificity per prognostic category, Kaplan-Meier curve and log rank test, hazard ratio (with 95% CI), median survival time	Kaplan-Meier curve and log rank test, hazard ratio (with 95% CI), median survival time	Only p-values reported<br><br>Results of statistical test stated as “significant” or “not significant” (without providing p-values) is considered as a particularly high risk of bias	No statistical test conducted (individual patient data provided)
Stratification / cut-off determination	Cut-off determined from ROC curve or scatterplots or taken from previous study	Cut-off based on mean / median / tertile	–	Cut-off determination not stated
Individual patient data provided?	Yes, outcome and mitotic count provided for each patient	Number of cases per outcome and prognostic cut-off provided (true negatives, true positives, false negative, false positive available)	–	No
**Overall risk of bias**	**Cumulative evaluation of all domains**			

Abbreviations: MC, mitotic count; MI, mitotic index, ROC, receiver operating characteristic; CI, confidence interval

We grouped the decision criteria of the risk of bias (RoB) into four domains: 1) study population, 2) outcome assessment, 3) mitotic activity measurement method, and 4) data analysis, upon which the overall RoB is based. The RoB was evaluated based on the credibility and comprehensibility of the published methods and results, as detailed in the table above. RoB evaluation was conducted regarding the information on mitotic activity only and other aspects of the articles were not evaluated. The decision criteria were developed based on previous recommendations [1-6] and were intended to be straightforward, applicable for this specific systematic review, and concede to the current practice of prognostic studies. We acknowledge that current recommendations for future studies might apply stricter criteria than we did in for this RoB evaluation. This was considered necessary to enable rating of low RoB in at least some studies. We acknowledge that this decision may result in underestimation of the true risks of bias in these categories and we suggest that these decision criteria are updated for future systematic reviews when several studies, that have adhered to these recommendations, have been published.

References

1. Boracchi P, Roccabianca P, Avallone G, Marano G. Kaplan-Meier Curves, Cox Model, and P-Values Are Not Enough for the Prognostic Evaluation of Tumor Markers: Statistical Suggestions for a More Comprehensive Approach. *Vet Pathol*. 2021;58**:** 795-808. 10.1177/03009858211014174
2. Donovan TA, Moore FM, Bertram CA, et al. Mitotic Figures-Normal, Atypical, and Imposters: A Guide to Identification. *Vet Pathol*. 2021;58**:** 243-257. 10.1177/0300985820980049
3. Krauth D, Woodruff TJ, Bero L. Instruments for assessing risk of bias and other methodological criteria of published animal studies: a systematic review. *Environ Health Perspect*. 2013;121**:** 985-992. 10.1289/ehp.1206389
4. Meuten DJ, Moore FM, Donovan TA, et al. International Guidelines for Veterinary Tumor Pathology: A Call to Action. *Vet Pathol*. 2021;58**:** 766-794. 10.1177/03009858211013712
5. Sauerbrei W, Taube SE, McShane LM, Cavenagh MM, Altman DG. Reporting Recommendations for Tumor Marker Prognostic Studies (REMARK): An Abridged Explanation and Elaboration. *J Natl Cancer Inst*. 2018;110**:** 803-811. 10.1093/jnci/djy088
6. Webster JD, Dennis MM, Dervisis N, et al. Recommended guidelines for the conduct and evaluation of prognostic studies in veterinary oncology. *Vet Pathol*. 2011;48**:** 7-18. 10.1177/0300985810377187

Supplemental material

Risk of bias

Supplemental Table S2. Risk of bias (⊕, low risk of bias; ○, moderate risk of bias; ⊖, high risk of bias) of the studies on the mitotic count (MC) based on four domains (D1-4: study population, outcome assessment, MC methods and data analysis).

Tumor type/group	(Sub)type / location	Article (year of publication)	Level of quality				
			D1: Study population	D2: Outcome assessment	D3: MC method	D4: Data analysis	Overall (D1-4)
Ceruminous gland tumor	Adenocarcinoma	Bacon et al. [1]	⊖	○	⊖	○	⊖
Hemangiosarcoma	Any location	Johannes et al. [13]	⊖	⊖	⊖	○	⊖
Lymphoma	Upper respiratory tract	Santagostino et al. [33]	○	○	⊖	○	○
Mammary tumor	Carcinomas	Dagher et al. [6]	⊕	○	⊕	○	○
	Carcinoma	Mills et al. [21]	○	○	⊕	○	○
	Carcinoma	Rosen et al. [27]	○	○	⊖	○	○
	Carcinoma	Weijer and Hart [36]	⊕	⊖	⊖	⊖	⊖
	Malignant	Weijer et al. [37]	⊕	⊖	⊖	⊖	⊖
Mast cell tumor	Cutaneous	Dobromylskyj et al. [8]	○	○	⊖	⊕	○
	Cutaneous, pleomorphic	Johnson et al. [14]	⊖	○	⊖	⊖	⊖
	Cutaneous	Lepri et al. [16]	○	○	⊖	⊖	⊖
	Cutaneous	Melville et al. [19]	○	○	⊖	⊖	⊖
	Cutaneous	Molander-McCrary et al. [22]	⊖	○	⊖	⊖	⊖

Tumor type/group	(Sub)type / location	Article (year of publication)	Level of quality				
			D1: Study population	D2: Outcome assessment	D3: MC method	D4: Data analysis	Overall (D1-4)
	Cutaneous	Sabattini and Bettini [28], 2019	⊕	⊕	○	⊕	⊕
	Cutaneous	Sabattini and Bettini [29], 2010	⊖	○	⊖	⊖	⊖
	Cutaneous	Sabattini et al. [31], 2013	○	○	⊖	○	○
	Intestinal	Sabattini et al. [30], 2016	⊖	○	⊖	○	⊖
Maxillary tumor	Any malignant tumor type	Liptak et al. [18]	⊖	○	⊖	○	⊖
Melanocytic tumor	Melanoma, non-ocular	Chamel et al. [3]	○	○	⊖	⊖	⊖
	Non-ocular	Pittaway et al. [23]	⊕	○	⊕	○	○
	Non-ocular	Sabattini et al. [32]	○	○	⊕	○	○
	Nasal planum	Reck and Kessler [25]	⊖	⊖	⊖	⊖	⊖
	Melanoma, iris	Wiggans et al. [38]	○	○	⊖	○	○
Merkel cell tumor	Carcinoma	Sumi et al. [35]	⊖	○	⊖	⊖	⊖
Oral tumors	Different tumor types initially suspected to be melanoma	Saverino et al. [34]	⊖	○	○	⊖	⊖
Osteosarcoma	any	Dimopoulou et al. [7]	○	○	○	⊖	⊖
Pancreatic tumor	Exocrine carcinoma	Linderman et al. [17]	⊖	○	⊖	⊖	⊖
Plasma cell tumor	Periarticular	Craig and Lieske [5]	⊖	⊖	○	⊖	⊖
Progressive histiocytosis	(sub)cutaneous	Coste et al. [4]	○	○	⊕	⊖	⊖
Salivary gland tumor	Malignant epithelial tumors	Hammer et al. [12]	⊖	⊖	⊖	○	⊖
Soft tissue sarcoma	Fibrosarcoma, skin, oral, bone	Bostock and Dye [2]	○	⊕	⊖	⊖	○
	Injection-site sarcoma	Guidice et al. [10]	⊕	⊖	⊖	○	⊖

Tumor type/group	(Sub)type / location	Article (year of publication)	Level of quality				
			D1: Study population	D2: Outcome assessment	D3: MC method	D4: Data analysis	Overall (D1-4)
	Injection-site fibrosarcoma	Kamenica et al. [15]	○	○	⊖	⊖	⊖
	Injection-site sarcoma	Porcellato et al. [24]	○	○	⊕	○	○
	Piloleiomyosarcoma	Guisado and Castro [11]	⊖	⊖	○	⊖	⊖
	(sub)cutaneous	Dobromylskyj et al. [9]	⊕	○	○	⊖	○
Squamous cell carcinoma	Oral	Yoshikawa et al. [39], 2016	○	⊕	○	⊖	⊖
	Oral	Yoshikawa et al.[40], 2012	○	○	○	⊖	⊖
	Spindle cell type, cutaneous	Rodriguez Guisado et al. [26]	⊖	○	○	⊖	⊖

Mitotic count methods

Supplemental Table S3. Summary of the mitotic count (MC) methods.

Tumor type/group	(Sub)type / location	Article, year of publication, and journal type	ROI location	Area exclusion	Enumerated HPF (mag.)	FN	FOV diameter	Area size*	Spatial arrangement of HPF	Other	MC value
Ceruminous gland tumor	Adenocarcinoma	Bacon et al. [1], 2002, NPFJ	N/A	N/A	1 (x 40)	N/A	N/A	N/A	NA	N/A	As counted
Hemangio-sarcoma	Any location	Johannes et al. [13], 2007, NPFJ	N/A	N/A	10 (400x)	N/A	N/A	N/A	N/A	N/A	As counted
Lymphoma	Upper respiratory tract	Santagostino et al. [33], 2015, PFJ	N/A	N/A	10 (400x)	N/A	N/A	N/A	N/A	Using the same microscope	As counted
Mammary tumor	Carcinomas	Dagher et al. [6], 2019, PFJ	Hotspot	N/A	10 (40x)	N/A	0.625 mm	N/A	Consecutive	N/A	As counted
	Carcinoma	Mills et al. [21], 2015, PFJ	Periphery and hotspot	N/A	10 (40x)	NA	0.53 mm	N/A	Consecutive	Care was taken to exclude apoptotic, pyknotic or otherwise hyper-chromatic nuclei.<br><br>Counts repeated if more than 20% difference between two observers	As counted
	Carcinoma	Rosen et al. [27], 2020, NPFJ	N/A	N/A	10 (N/A)	N/A	N/A	N/A	Consecutive	N/A	As counted
	Carcinoma	Weijer and Hart [36], 1983, NPFJ	N/A	N/A	1 (N/A)	N/A	N/A	N/A	N/A	N/A	As counted
	Malignant	Weijer et al. [37], 1972, NPFJ	N/A	N/A	1 (N/A)	N/A	N/A	N/A	N/A	Mitotic and hyperchromatic figures	As counted
Mast cell tumor	Cutaneous	Dobromylskyj et al. [8], 2015, PFJ	N/A	N/A	10 (x400)	N/A	N/A	N/A	N/A	N/A	As determined for previous study [19]
	Cutaneous, pleomorphic	Johnson et al. [14], 2002, PFJ	N/A	N/A	10 (N/A)	N/A	N/A	N/A	N/A	N/A	As counted
	Cutaneous	Lepri et al. [16], 2003, NPFJ	N/A	N/A	10 (N/A)	N/A	N/A	N/A	N/A	N/A	As counted
	Cutaneous	Melville et al. [19], 2015, NPFJ	N/A	N/A	10 (x400)	N/A	N/A	N/A	N/A	N/A	As counted
	Cutaneous	Molander-McCrary et al. [22], 1998, NPFJ	N/A	N/A	At least 10 (400x)	N/A	N/A	N/A	N/A	N/A	Per 1 HPF
	Cutaneous	Sabattini and Bettini [28], 2019, PFJ	Hotspot	N/A	10 (400x)	22 mm	N/A	2.37 mm²	Consecutive, non-overlapping	N/A	As counted
	Cutaneous	Sabattini and Bettini [29], 2010, PFJ	N/A	N/A	10 (400x)	N/A	N/A	N/A	N/A	N/A	As counted
	Cutaneous	Sabattini et al. [31], 2013, PFJ	Hotspot	N/A	10 (400x)	N/A	N/A	N/A	N/A	N/A	As counted
	Intestinal	Sabattini et al. [30], 2016, NPFJ	Hotspot	N/A	10 (x400)	N/A	N/A	N/A	N/A	N/A	As counted
Maxillary tumor	Any malignant tumor type	Liptak et al. [18], 2021, NPFJ	N/A	N/A	N/A	N/A	N/A	N/A	N/A	Data taken from reports	As counted?

Tumor type/group	(Sub)type / location	Article, year of publication, and journal type	ROI location	Area exclusion	Enumerated HPF (mag.)	FN	FOV diameter	Area size*	Spatial arrangement of HPF	Other	MC value
Melanocytic tumor	Melanoma, non-ocular	Chamel et al. [3], 2017, NPFJ	N/A	N/A	N/A	N/A	N/A	N/A	N/A	Data taken from reports	Low, moderate, high
	Non-ocular	Pittaway et al. [23], 2019, PFJ	Hotspot	Ulceration and necrosis	10 (400x)	N/A	N/A	2.37 mm²	Consecutive, non-overlapping	Bleached sections, if required	As counted
	Non-ocular	Sabattini et al. [32], 2018, NPFJ	Hotspot	Necrosis, inflammation	10 (400x)	22 mm	N/A	2.37 mm²	Consecutive, non-overlapping	Bleached sections for pigmented tumors (>25% cells)	As counted
	Nasal planum	Reck and Kessler [25], 2021, NPFJ	N/A	N/A	10 (N/A)	N/A	N/A	N/A	N/A	Bleached sections, if required	As counted
	Melanoma, iris	Wiggans et al. [38], 2016, NPFJ	N/A	N/A	10 (x400)	N/A	N/A	N/A	N/A	N/A	As counted
Merkel cell tumor	Carcinoma	Sumi et al. [35], 2018, NPFJ	N/A	N/A	10 (x400)	N/A	N/A	N/A	N/A	N/A	Per 1 HPF
Oral tumors	Different tumor types initially suspected to be melanoma	Saverino et al. [34], 2021, PFJ	N/A	N/A	10 (N/A)	N/A	N/A	2.37 mm²	N/A	N/A	As counted
Osteo-sarcoma	any	Dimopoulou et al. [7], 2008, NPFJ	Random	N/A	3 (x400)	N/A	N/A	N/A	N/A	N/A	As counted
Pancreatic tumor	Exocrine carcinoma	Linderman et al. [17], 2013, NPFJ	N/A	N/A	N/A	N/A	N/A	N/A	N/A	Data taken from medical records	Per 1 HPF
Plasma cell tumor	Periarticular	Craig and Lieske [5], 2022, PFJ	N/A	N/A	10	N/A	N/A	2.37 mm²	N/A	N/A	As counted
Progressive histiocytosis	(sub)cutaneous	Coste et al. [4], 2019, PFJ	Random	N/A	10	N/A	N/A	2.37 mm²	N/A	N/A	Per 1 HPF
Salivary gland tumor	Malignant epithelial tumors	Hammer et al. [12], 2001, NPFJ	N/A	N/A	1 (40x)	N/A	N/A	N/A	N/A	N/A	As counted
Soft tissue sarcoma	Fibrosarcoma, skin, oral, bone	Bostock and Dye [2], 1979, NPFJ	N/A	Necrotic area	10 (400)	N/A	N/A	N/A	N/A	N/A	As counted
	Injection-site sarcoma	Guidice et al. [10], 2010, NPFJ	N/A	N/A	10 (400x)	N/A	N/A	N/A	N/A	N/A	Per 1 HPF
	Injection-site fibrosarcoma	Kamenica et al. [15], 2008,NPFJ	N/A	N/A	15 (40x)	N/A	N/A	N/A	N/A	N/A	As counted
	Injection-site sarcoma	Porcellato et al. [24], 2017, PFJ	Hotspot	Necrosis, inflammation	10 (40x)	22	N/A	2.37 mm²	Contiguous	N/A	Mean value of three observers
	Piloleiomyosarcoma	Guisado and Castro [11], 2022, PFJ	N/A	N/A	30 (400x)	22 mm	0.55 mm	N/A	N/A	N/A	As counted
	(sub)cutaneous	Dobromylskyj et al. [9], 2021, NPFJ	N/A	N/A	10 (x400)	N/A	N/A	2.37 mm²	N/A	N/A	As counted
Squamous cell carcinoma	Oral	Yoshikawa et al. [39], 2016, NPFJ	Random	N/A	2 x 10 (400x)	N/A	N/A	N/A	N/A	Counted twice by a single author	Per 1 HPF (mean and maximum)
	Oral	Yoshikawa et al.[40], 2012, NPFJ	Random	N/A	2 x 10 (400x)	N/A	N/A	N/A	N/A	Counted twice by a single author	Per 1 HPF (mean and maximum)
	Spindle cell type, cutaneous	Rodriguez Guisado et al. [26], 2021, PFJ	N/A	N/A	10 (400x)	N/A	0.55 mm	2.37 mm²	N/A	N/A	Median per 10 PHF and standard deviation

Abbreviations: PFJ, pathology focused journal; NPFJ, non-pathology focused journal; ROI, region of interest; mag., magnification; HPF, high power fields; FN, field number of the microscope; FOV, field of view; N/A, not available

* For studies that provided the field number or the field of view diameter of their microscope, we used the formula provided by Meuten et al. [20] to calculate the area size in mm².

Summary text of Supplemental Table S3:

Of the 36/39 studies with described MC methods (data not taken from medical records), the **selected ROI** within the tumor section were hotspots (high mitotic activity, N = 7 [6,23,24,28,30-32]), hotspots in the periphery of the tumor (N = 1 [21]) or random areas (N = 4 [4,7,39,40]). The selected ROI was not specified in the remaining 24/36 (67%) studies, which comprised 73% (N = 16/22) of the publications before 2017 and 57% (N = 8/14) after 2016 as well as 76% (N = 16/21) of the publications in non-pathology focused and 53% (N = 5/15) of the pathology focused journals. Four studies specified that they avoided necrotic areas and ulceration or inflammation when selecting the ROIs.[2,23,24,32]

The number of **enumerated HPFs** were indicated in the 36 studies as 1 (N = 4, 11%), 3 (N = 1, 3%), 10 (N = 26, 72%), two times 10 (N = 2, 6%), at least 10 (N = 1, 3%), 15 (N = 1, 3%), or 30 (N = 1, 3%), while 93% (N = 14/15) of the articles published in pathology focused journals after 2016 used 10 HPFs. The used magnification of a HPF was indicated as 400x in 27 studies and not reported in 9. In eight studies, the MC values used for calculation of prognostic value differed from the values counted: one study in which 30 HPFs were enumerated used the MC value per 10 HPFs,[11] four studies in which (at least) 10 HPFs were enumerated used the MC value per 1 HPF [4,10,22,35], one study in which 10 HPFs were enumerated used the “median per 10 HPF ± standard deviation” [26], and two studies in which 10 HPFs were enumerated twice used the mean or maximum MC value per one HPF.[39,40]

The precise **area size** enumerated is indicated in 12/36 (33%) publications either by stating the field number of the microscope (N = 4), the diameter of the field of view of a HPF (N = 4), or/and by the area size (in mm^2) of the enumerated HPFs (N = 9). Of these 12 articles, 11 were published after 2016 and 10 in pathology focused journals. The area size was 2.21 mm^2 (N = 1), 2.37 mm^2 (N = 10), and 3.07 mm^2 (N = 1).

The **spatial arrangement** of the individual HPFs is described in 6/36 (17%) studies as consecutive / contiguous (N = 3) or consecutive non-overlapping (N = 3); five of these articles (83%) are published in pathology focused journals and after 2016.

Prognostic value: Survival

Supplemental Table S4. Summary of the prognostic value of the mitotic count (MC) with regard to survival time and survival rate including all-cause mortality (ACM) and tumor-related mortality (TRM).

Tumor type/group	(Sub)type / location	Article	Number of cases / tumors with outcome	ACM / TRM	IPD	Cut-off ranges	MST low MC value in days (d), weeks (w), or months (m	MST high MC value in days (d) or weeks (w)	Kaplan-Meier curve	Log rank test	Cox proportional hazard regression model, univariable	ROC and AUC	Other
Ceruminous gland tumor	Adenocarcinoma	Bacon et al. [1]	11	?	-	≤ 2, ≥ 3	-	-	Yes	p = 0,036	-	-	-
Hemangio-sarcoma	Any location	Johannes et al. [13]	15	?	-	≤3, >3	Could not be calculated due to excessive censoring	60 d	-	p = 0.020	-	-	TP: 8, TN: 5, FP: 1, FN: 1,<br><br>Sen: 89%, Spe: 83%, Acc: 86% *
Lymphoma	Upper respiratory tract	Santagostino et al. [33]	39	ACM	-	-	-	-	-	-	HR = 1.018, 95%CI: 0.9911 – 1.046, p = 0.191	-	Multivariable HR = 1.0108, 95%CI: 0.9765 – 1.0463; p = 0.5412
Mammary tumor	Carcinomas	Dagher et al. [6]	342	ACM	-	≤ 33, > 33	400 d	332 d	-	p = 0.05	HR (low MC cases)= 0.79, 95%CI: 0.63 – 0.99<br><br>HR (high grade cases)*: 1.27, 95%CI: 1.01 – 1.59	-	-
				TRM	-	≤ 33, > 33	-	-	-	p = 0.13	HR (low MC cases) = 0.81 95%CI: 0.61 – 1.06<br><br>HR (high MC cases)*: 1.24, 95%CI: 0.94 – 1,64	-	-
	Carcinoma	Mills et al. [21]	97 / 108	TRM	-	≤62, >62	18 m	9 m	-	p = 0.021	HR = 0.59, 95%CI: 0.37 – 0.92<br><br>HR (reference group reversed)*: 1.69, 95%CI: 1.08 – 3.13	-	Multivariable analysis: HR = 1.96, 95%CI: 1.23 – 3.12, p = 0.004

Tumor type/group	(Sub)type / location	Article	Number of cases / tumors with outcome	ACM / TRM	IPD	Cut-off ranges	MST low MC value in days (d), weeks (w), or months (m	MST high MC value in days (d) or weeks (w)	Kaplan-Meier curve	Log rank test	Cox proportional hazard regression model, univariable	ROC and AUC	Other
	Carcinoma	Rosen et al. [27]	30	ACM	-	-	-	-	Yes	-	continuous values: HR = 1,025, 95%CI: 1,012 - 1,037, p < 0,001 (Table 1); categorial values: HR = 4.484, 95%CI 1,758 - 11,262, p = 0,001	-	-
	Carcinoma	Weijer and Hart [36]	202	?	-	1, 1-2, 2, 2-3, 3	-	-	Yes	p = 0.017	-	-	-
	Malignant	Weijer et al. [37]	37	?	-	Low, moderate, high	-	-	-	-	-	-	p < 0,005 (unknown test)
Mast cell tumor	Cutaneous	Dobromylskyj et al. [8]	57 / 71 (same cases as in [19])	TRM	-	<5, ≥5	1696 d (95%CI: 1546 - 1846)	1150 d (95%CI: 783 - 1517	Yes	p = 0.01	-	Yes; AUC = 0,79; 95%CI: 0,61 - 0,96	Sen: 60%<br>Spe: 80%<br>Mann-Whitney U Test: p = 0,004
	Cutaneous, pleomorphic	Johnson et al. [14]	-	-	-	-	-	-	-	-	-	-	-
	Cutaneous	Lepri et al. [16]	-	-	-	-	-	-	-	-	-	-	-
	Cutaneous	Melville et al. [19]	69 / 86	TRM	-	-	-	-	-	-	-	-	non-parameteric Wilcoxon rank-sum test: p < 0.002
	Cutaneous	Molander-McCrary et al. [22]	32	TRM	-	0, 1, 2-3, >3	-	-	-	NS	-	-	-
	Cutaneous	Sabattini and Bettini [28], 2019	63	TRM within 1000d	-	≤5, >5	-	-	-	-	-	AUC = 0.92, 95%CI: 0.86-0.99; p < 0.001	analysis of variance and chi-square/Fisher's exact test: p = 0.002<br><br>Odds ratio: 53.2, 95%CI: 6.23-454.44<br><br>Sen: 100%, Spe: 79%
	Cutaneous	Sabattini and Bettini [29], 2010	23	TRM	-	-	`-	-	-	-	-	-	Fisher exact test or Kruskal–Wallis test for parametric analysis of

Tumor type/group	(Sub)type / location	Article	Number of cases / tumors with outcome	ACM / TRM	IPD	Cut-off ranges	MST low MC value in days (d), weeks (w), or months (m	MST high MC value in days (d) or weeks (w)	Kaplan-Meier curve	Log rank test	Cox proportional hazard regression model, univariable	ROC and AUC	Other
													variance: p = 0.0024
	Cutaneous	Sabattini et al. [31], 2013	24 / 34	?	-	0-5, >5	-	-	-	-	HR = 13.097, 95%CI: 1.580 – 108.586, p = 0.017	-	Multivariable HR = 8.98, 95%CI: 1.04-77.849, p = 0.46
	Intestinal	Sabattini et al. [30], 2016	11	?	Yes	0-2, >2	-	-	-	p = 0.012	-	-	TP: 4, TN: 3, FP: 0, FN: 4, Sen: 50%, Spe: 100%, Acc: 63.6% *
Maxillary tumor	Any malignant tumor type	Liptak et al. [18]	29	TRM	-	-	-	-	-	-	HR: 1.12, 95%CI: 1.01 – 1.24, p = 0.03	-	-
Melanocytic tumor	Melanoma, non-ocular	Chamel et al. [3]	26	ACM?	Yes	Low, modera te, high	-	-	-	NS	-	-	-
	Non-ocular	Pittaway et al. [23]	79	TRM	-	<4, ≥4	-	-	Yes	-	p < 0,001	-	-
	Non-ocular	Sabattini et al. [32]	33	?	-	0-5, >5	689 d (95%CI: 0 - 1451)	119 d (95%CI: 32 - 206)	-	0.013	-	-	Multivariable HR = 6.77, 95%CI: 0.36-128.02, p = 0.202
	Nasal planum	Reck and Kessler [25]	4	ACM	Yes	-	-	-	-	-	-	-	-
	Melanoma, iris	Wiggans et al. [38]	-	-	-	-	-	-	-	-	-	-	-
Merkel cell tumor	Carcinoma	Sumi et al. [35]	17	ACM	Yes	-	-	-	-	-	-	-	-
Oral tumors	Different tumor types initially suspected to be melanoma	Saverino et al. [34]	14	ACM	Yes	-	-	-	-	-	-	-	-
Osteo-sarcoma	any	Dimopoulou et al. [7]	-	-	-	-	-	-	-	-	-	-	-
Pancreatic tumor	Exocrine carcinoma	Linderman et al. [17]	30	ACM	-	-	-	-	-	-	NS	-	-
Plasma cell tumor	Periarticular	Craig and Lieske [5]	7	?	Yes	-	-	-	-	-	-	-	-
Progressive histiocytosis	(sub)cutaneous	Coste et al. [4]	19	ACM?	-	-	-	-	-	NS	-	-	-
Salivary gland tumor	Malignant epithelial tumors	Hammer et al. [12]	30	?	-	-	-	-	Yes, lower MC had shorter survival	p = 0,003	-	-	-
-Soft tissue sarcoma	Fibrosarcoma, skin, oral, bone	Bostock and Dye [2]	35	?	-	≤5, >5	128 w	16 w		p < 0.02	-	-	-
	Injection-site sarcoma	Guidice et al. [10]	-	-	-	-	-	-	-	-	-	-	-
	Injection-site fibrosarcoma	Kamenica et al. [15]	41	ACM	Yes	0-10, 11-33, >33	-	-	-	NS	-	-	-

Tumor type/group	(Sub)type / location	Article	Number of cases / tumors with outcome	ACM / TRM	IPD	Cut-off ranges	MST low MC value in days (d), weeks (w), or months (m	MST high MC value in days (d) or weeks (w)	Kaplan-Meier curve	Log rank test	Cox proportional hazard regression model, univariable	ROC and AUC	Other
				TRM*	Yes	0-10, 11-33, >33	-	-	-	-	-	-	MC 0-10: 7/14 MC 11-33: 11/14 MC >33: 10/13 *
	Injection-site sarcoma	Porcellato et al. [24]	24	TRM	-	<20, ≥20	Not reached	994 d	Yes	p < 0,016	HR = 1.07; 95%CI 1.01 – 1.14; p = 0.017	-	-
	Piloleiomyosarcoma	Guisado and Castro [11]	-	-	-	-	-	-	-	-	-	-	-
	(sub)cutaneous	Dobromylskyj et al. [9]	47	TRM	-	-	-	-	-	-	-	-	Mann-Whitney U Test: p = 0.00362
Squamous cell carcinoma	Oral	Yoshikawa et al. [39], 2016	20	TRM	-	-	-	-	-	NS	-	-	-
	Oral	Yoshikawa et al.[40], 2012	22	ACM?	-	-	-	-	-	NS	-	-	-
	Spindle cell type, cutaneous	Rodriguez Guisado et al. [26]	18	TRM	Yes	<14, ≥14	-	-	-	-	-	-	TP: 2, TN: 14, FP: 2, FN: 0; Sen: 100%, Spe: 50%, Acc: 88% *

Abbreviations: ACM, all-cause mortality; TRM, tumor-related mortality; IPD, individual patient data; MST, median survival time; -, not available; NS, not significant (p > 0.05) if actual p-value is not provided in the manuscript; HR, hazard ratio; ROC, receiver operating characteristic curve; AUC, area under the ROC curve; TP, true positives; TN, true negatives; FP, false positives; FN, false negatives; Sen, sensitivity; Spe, specificity; Acc, accuracy; * calculated from individual patient data and proposed cut-off ranges (TP, TN, FP, FN, Spe, Sen) or mathematic formula (HR with inversed reference group = 1 / hazard ratio)

Prognostic value: Progression, metastasis and recurrence

Supplemental Table S5. Summary of the prognostic value of the mitotic count (MC) with regard to progression (Pro, recurrence, metastasis, and/or death), recurrence (Rec), and metastasis (Met).

Tumor type/group	(Sub)type / location	Article	Number of cases with outcome	Outcome metric	IPD	Cut-off ranges	Kaplan-Meier curve	Log rank test	Cox proportional hazard regression model, univariable	ROC, AUC	Other
Ceruminous gland tumor	Adenocarcinoma	Bacon et al. [1]	-	-	-	-	-	-	-		-
Hemangio-sarcoma	Any location	Johannes et al. [13]	-	-	-	-	-	-	-		-
Lymphoma	Upper respiratory tract	Santagostino et al. [33]	-	-	-	-	-	-	-		-
Mammary tumor	Carcinomas	Dagher et al. [6]	-	-	-	-	-	-	-		-
	Carcinoma	Mills et al. [21]	-	-	-	-	-	-	-		-
	Carcinoma	Rosen et al. [27]	30	Pro	-	-	-	-	HR = 1.021, 95%CI 1.008 – 1.035, p = 0.002		-
	Carcinoma	Weijer and Hart [36]	202	Rec	-	1, 1-2, 2, 2-3, 3	-	-	-	-	NS
	Malignant	Weijer et al. [37]	-	-	-	-	-	-	-		-
Mast cell tumor	Cutaneous	Dobromylskyj et al. [8]	-	-	-	-	-	-	-		-
	Cutaneous, pleomorphic	Johnson et al. [14]	14	Rec	Yes	<1, 1-2	-	-	-	-	TP: 1, TN: 13, FP: 0, FN: 0, Sen: 100%, Spe: 100% *
	Cutaneous	Lepri et al. [16]	30	Pro: Rec, Met, and cutaneous dissemination	-	0-3, >4	-	-	-	-	Outcome rate:<br>Low MC: 8/23<br>High MC: 5/7<br>TP: 5, TN: 15, FP: 2, FN: 8, Sen: 38%, Spe: 88%, Acc: 67% *
	Cutaneous	Melville et al. [19]	-	-	-	-	-	-	-		-
	Cutaneous	Molander-McCrary et al. [22]	-	-	-	-	-	-	-		-
	Cutaneous	Sabattini and Bettini [28], 2019	-	-	-	-	-	-	-		-
	Cutaneous	Sabattini and Bettini [29], 2010	-	-	-	-	-	-	-		-
	Cutaneous	Sabattini et al. [31], 2013	24 / 34	Pro	-	0-5, >5	-	-	HR = 3.725, 95%CI: 1.1 – 12.617, p = 0.035		-
	Intestinal	Sabattini et al. [30], 2016	-	-	-	-	-	-	-		-
Maxillary tumor	Any malignant tumor type	Liptak et al. [18]	29	Pro	-	-	-	-	HR: 1.11, 95%CI: 1.02 – 1.21; p = 0.02		-
Melanocytic tumor	Melanoma, non-ocular	Chamel et al. [3]	29	Met	Yes	Low, moderate, high	-	-	-	-	Metastasis rate:<br>Low MC: 0/2<br>Moderate MC: 0/6<br>High MC: 1/21
	Non-ocular	Pittaway et al. [23]	-	-	-	-	-	-	-		-
	Non-ocular	Sabattini et al. [32]	-	-	-	-	-	-	-		-

Tumor type/group	(Sub)type / location	Article	Number of cases with outcome	Outcome metric	IPD	Cut-off ranges	Kaplan-Meier curve	Log rank test	Cox proportional hazard regression model, univariable	ROC, AUC	Other
	Nasal planum	Reck and Kessler [25]	4	Pro	Yes	-	-	-	-		-
	Melanoma, iris	Wiggans et al. [38]	47	Met	-	0-7, >7	Yes	p = 0.024	-		-
Merkel cell tumor	Carcinoma	Sumi et al. [35]	14	Pro	Yes	-	-	-	-		-
				Met	Yes	-	-	-	-	-	-
Oral tumors	Different tumor types initially suspected to be melanoma	Saverino et al. [34]	14	Met	Yes	-	-	-	-		-
Osteo-sarcoma	any	Dimopoulou et al. [7]	43	Rec	-	0, 1, >1	-	-	-		Multivariate HR: 1.24, 95%CI: 1.0 – 1.5; p = 0.037
Pancreatic tumor	Exocrine carcinoma	Linderman et al. [17]	30	Pro	-	-	-	-	NS		-
Plasma cell tumor	Periarticular	Craig and Lieske [5]	5	Met	Yes	-	-	-	-		-
Progressive histiocytosis	(sub)cutaneous	Coste et al. [4]	-	-	-	-	-	-	-		-
Salivary gland tumor	Malignant epithelial tumors	Hammer et al. [12]	-	-	-	-	-	-	-		-
Soft tissue sarcoma	Fibrosarcoma, skin, oral, bone	Bostock and Dye [2]	35	Rec	-	≤5, >5	-	-	-	-	Low MC: 12/19<br>High MC: 12/16<br>chi-square test: NS
	Injection-site sarcoma	Guidice et al. [10]	48	Rec	-	<1, ≥1-<2, ≥2	-	-	-	-	Crude odds ratio (baseline: MC <1): MC ≥1-<2: 0.0673, 95%CI: 1.6514, p = 0.1714;<br>MC ≥2: 0.0901, 95%CI: 1.4154, p = 0.1428
	Injection-site fibrosarcoma	Kamenica et al. [15]	41	Rec	Yes	0-10, 11-33, >33	-	NS	-		MC 0-10: 5/11<br>MC 11-33: 8/12<br>MC >33: 7/12 *
	Injection-site sarcoma	Porcellato et al. [24]	24	Rec	-	0-9, 10-19, >19	-	-	HR = 1.09, 95%CI: 1.04 – 1.14, p = 0.001	Yes; AUC = 0.882, 95%CI: 0.747 – 1.000, p = 0.002	MC 0-9 = 0/6, MC 10-19 = 3/9, MC >19 = 7/9; Fisher exact test: 0,008; Sen: 70%, Spe: 86%, Acc: 80%
	Piloleiomyosarcoma	Guisado and Castro [11]	9	Rec	Yes	≤12, >12	-	-	-	-	TP: 2, TN: 6, FP: 1, FN: 0, Sen: 100%, Spe: 86%, Acc: 89% *
				Met	Yes	≤12, >12	-	-	-	-	TP: 1, TN: 6, FP: 2, FN: 0, Sen: 100%, Spe: 75%, Acc: 78% *
	(sub)cutaneous	Dobromylskyj et al. [9]	-	-	-	-	-	-	-		-
Squamous cell carcinoma	Oral	Yoshikawa et al. [39], 2016	20	Pro	-	-	-	NS	-		-
	Oral	Yoshikawa et al.[40], 2012	-	-	-	-	-	-	-		-
	Spindle cell type, cutaneous	Rodriguez Guisado et al. [26]	18	Met and Rec	Yes	<14, ≥14	-	-	-	-	Analysis of variance: p < 0,05<br><br>TP: 4, TN: 14, FP: 0, FN: 0; Sen: 100%, Spe: 100% *

Abbreviations: PFI, progression-free interval; DFI, disease-free interval; RFI, recurrence-free interval; TRD, tumor-related disease; MST, median survival time; -, not available; NS, not significant (p > 0.05)) if actual p-value is not provided in the manuscript; HR,

hazard ratio; ROC, receiver operating characteristic curve; AUC, area under the ROC curve; TP, true positives; TN, true negatives; FP, false positives; FN, false negatives; Sen, sensitivity; Spe, specificity; Acc, accuracy; * calculated from individual patient data and proposed cut-off ranges

References

1. Bacon NJ, Gilbert RL, Bostock DE, White RAS. Total ear canal ablation in the cat: Indications, morbidity and long-term survival. *Journal of Small Animal Practice*. 2003;44**:** 430-434.
2. Bostock DE, Dye MT. Prognosis after surgical excision of fibrosarcomas in cats. *J Am Vet Med Assoc*. 1979;175**:** 727-728.
3. Chamel G, Abadie J, Albaric O, Labrut S, Ponce F, Ibisch C. Non-ocular melanomas in cats: a retrospective study of 30 cases. *Journal of Feline Medicine and Surgery*. 2017;19**:** 351-357.
4. Coste M, Prata D, Castiglioni V, et al. Feline progressive histiocytosis: a retrospective investigation of 26 cases and preliminary study of Ki67 as a prognostic marker. *J Vet Diagn Invest*. 2019;31**:** 801-808. 10.1177/1040638719884950
5. Craig LE, Lieske DE. Periarticular plasma cell tumors in cats. *Vet Pathol*. 2022;59**:** 264-268. 10.1177/03009858211048621
6. Dagher E, Abadie J, Loussouarn D, Campone M, Nguyen F. Feline Invasive Mammary Carcinomas: Prognostic Value of Histological Grading. *Vet Pathol*. 2019;56**:** 660-670.
7. Dimopoulou M, Kirpensteijn J, Moens H, Kik M. Histologic prognosticators in feline osteosarcoma: A comparison with phenotypically similar canine osteosarcoma. *Veterinary Surgery*. 2008;37**:** 466-471.
8. Dobromylskyj MJ, Rasotto R, Melville K, Smith KC, Berlato D. Evaluation of Minichromosome Maintenance Protein 7 and c-KIT as Prognostic Markers in Feline Cutaneous Mast Cell Tumours. *Journal of Comparative Pathology*. 2015;153**:** 244-250.
9. Dobromylskyj MJ, Richards V, Smith KC. Prognostic factors and proposed grading system for cutaneous and subcutaneous soft tissue sarcomas in cats, based on a retrospective study. *Journal of Feline Medicine and Surgery*. 2021;23**:** 168-174.
10. Giudice C, Stefanello D, Sala M, et al. Feline injection-site sarcoma: recurrence, tumour grading and surgical margin status evaluated using the three-dimensional histological technique. *Vet J*. 2010;186**:** 84-88. 10.1016/j.tvjl.2009.07.019
11. Guisado FR, Castro PL. Piloleiomyosarcoma in cats: Histological and immunohistochemical features. *Vet Pathol*. 2022;59**:** 57-62.
12. Hammer A, Getzy D, Ogilvie G, Upton M, Klausner J, Kisseberth WC. Salivary gland neoplasia in the dog and cat: survival times and prognostic factors. *J Am Anim Hosp Assoc*. 2001;37**:** 478-482. 10.5326/15473317-37-5-478
13. Johannes CM, Henry CJ, Turnquist SE, et al. Hemangiosarcoma in cats: 53 cases (1992-2002). *Journal of the American Veterinary Medical Association*. 2007;231**:** 1851-1856.
14. Johnson TO, Schulman FY, Lipscomb TP, Yantis LD. Histopathology and biologic behavior of pleomorphic cutaneous mast cell tumors in fifteen cats. *Vet Pathol*. 2002;39**:** 452-457. 10.1354/vp.39-4-452
15. Kamenica K, Brill T, Hirschberger J, Köstlin R, Hermanns W. Vakzineassoziierte feline Fibrosarkome: histopathologische Befunde und Pognose. *Tierarztl Prax*. 2008;36**:** 135-141.
16. Lepri E, Ricci G, Leonardi L, Sforna M, Mechelli L. Diagnostic and prognostic features of feline cutaneous mast cell tumours: a retrospective analysis of 40 cases. *Vet Res Commun*. 2003;27 Suppl 1**:** 707-709. 10.1023/b:verc.0000014253.07296.0c
17. Linderman MJ, Brodsky EM, de Lorimier LP, Clifford CA, Post GS. Feline exocrine pancreatic carcinoma: a retrospective study of 34 cases. *Vet Comp Oncol*. 2013;11**:** 208-218. 10.1111/j.1476-5829.2012.00320.x

18. Liptak JM, Thatcher GP, Mestrinho LA, et al. Outcomes of cats treated with maxillectomy: 60 cases. A Veterinary Society of Surgical Oncology retrospective study. *Veterinary and comparative oncology*. 2021;19**:** 641-650.
19. Melville K, Smith KC, Dobromylskyj MJ. Feline cutaneous mast cell tumours: a UK-based study comparing signalment and histological features with long-term outcomes. *Journal of Feline Medicine and Surgery*. 2015;17**:** 486-493.
20. Meuten DJ, Moore FM, George JW. Mitotic Count and the Field of View Area: Time to Standardize. *Vet Pathol*. 2016;53**:** 7-9. 10.1177/0300985815593349
21. Mills SW, Musil KM, Davies JL, et al. Prognostic Value of Histologic Grading for Feline Mammary Carcinoma: A Retrospective Survival Analysis. *Vet Pathol*. 2015;52**:** 238-249.
22. Molander-McCrary H, Henry CJ, Potter K, Tyler JW, Buss MS. Cutaneous mast cell tumors in cats: 32 cases (1991-1994). *J Am Anim Hosp Assoc*. 1998;34**:** 281-284. 10.5326/15473317-34-4-281
23. Pittaway R, Dobromylskyj MJ, Erles K, et al. Nonocular Melanocytic Neoplasia in Cats: Characterization and Proposal of a Histologic Classification Scheme to More Accurately Predict Clinical Outcome. *Vet Pathol*. 2019;56**:** 868-877.
24. Porcellato I, Menchetti L, Brachelente C, et al. Feline Injection-Site Sarcoma: Matrix Remodeling and Prognosis. *Vet Pathol*. 2017;54**:** 204-211.
25. Reck A, Kessler M. Melanocytic tumours of the nasal planum in cats: 10 cases (2004-2019). *J Small Anim Pract*. 2021;62**:** 131-136. 10.1111/jsap.13265
26. Rodríguez Guisado F, Suárez-Bonnet A, Ramírez GA. Cutaneous Spindle Cell Squamous Cell Carcinoma in Cats: Clinical, Histological, and Immunohistochemical Study. *Vet Pathol*. 2021;58**:** 503-507.
27. Rosen S, Brisson BK, Durham AC, et al. Intratumoral collagen signatures predict clinical outcomes in feline mammary carcinoma. *PloS one*. 2020;15**:** e0236516. 10.1371/journal.pone.0236516
28. Sabattini S, Bettini G. Grading Cutaneous Mast Cell Tumors in Cats. *Vet Pathol*. 2019;56**:** 43-49. 10.1177/0300985818800028
29. Sabattini S, Bettini G. Prognostic value of histologic and immunohistochemical features in feline cutaneous mast cell tumors. *Vet Pathol*. 2010;47**:** 643-653.
30. Sabattini S, Giantin M, Barbanera A, et al. Feline intestinal mast cell tumours: clinicopathological characterisation and KIT mutation analysis. *Journal of Feline Medicine and Surgery*. 2016;18**:** 280-289.
31. Sabattini S, Guadagni Frizzon M, Gentilini F, Turba ME, Capitani O, Bettini G. Prognostic significance of Kit receptor tyrosine kinase dysregulations in feline cutaneous mast cell tumors. *Vet Pathol*. 2013;50**:** 797-805. 10.1177/0300985813476064
32. Sabattini S, Renzi A, Albanese F, et al. Evaluation of Ki-67 expression in feline non-ocular melanocytic tumours. *BMC Veterinary Research*. 2018;14.
33. Santagostino SF, Mortellaro CM, Boracchi P, et al. Feline upper respiratory tract lymphoma: site, cyto-histology, phenotype, FeLV expression, and prognosis. *Vet Pathol*. 2015;52**:** 250-259. 10.1177/0300985814537529
34. Saverino KM, Durham AC, Kiupel M, Reiter AM. Immunohistochemical evaluation of suspected oral malignant melanoma in cats. *Vet Pathol*. 2021;58**:** 1017-1024. 10.1177/03009858211025793
35. Sumi A, Chambers JK, Doi M, Kudo T, Omachi T, Uchida K. Clinical features and outcomes of Merkel cell carcinoma in 20 cats. *Veterinary and comparative oncology*. 2018;16**:** 554-561.
36. Weijer K, Hart AA. Prognostic factors in feline mammary carcinoma. *J Natl Cancer Inst*. 1983;70**:** 709-716.
37. Weijer K, Head KW, Misdorp W, Hampe JF. Feline malignant mammary tumors. I. Morphology and biology: some comparisons with human and canine mammary carcinomas. *J Natl Cancer Inst*. 1972;49**:** 1697-1704. 10.1093/jnci/49.6.1697

38. Wiggans KT, Reilly CM, Kass PH, Maggs DJ. Histologic and immunohistochemical predictors of clinical behavior for feline diffuse iris melanoma. *Veterinary Ophthalmology*. 2016;19**:** 44-55.
39. Yoshikawa H, Ehrhart EJ, Charles JB, Custis JT, Larue SM. Assessment of predictive molecular variables in feline oral squamous cell carcinoma treated with stereotactic radiation therapy. *Veterinary and comparative oncology*. 2016;14**:** 39-57.
40. Yoshikawa H, Ehrhart EJ, Charles JB, Thamm DH, Larue SM. Immunohistochemical characterization of feline oral squamous cell carcinoma. *Am J Vet Res*. 2012;73**:** 1801-1806.

Supplemental Table S6. Summary of each evaluated study regarding the prognostic significance of the mitotic count in different feline tumor types based on the p-value approach or conclusion by the authors.

Tumor type	Reference (author and year of publication)	Association suggested with			
		Survival	Progression	Metastasis	Recurrence
Ceruminous gland adenocarcinoma	Bacon et al., 2003	Yes	–	–	–
Hemangiosarcoma	Johannes et al., 2007	Yes	–	–	–
Lymphoma	Santagostino et al., 2015	No	–	–	–
Mammary tumor	Dagher et al., 2019	Yes	–	–	–
	Mills et al., 2015	Yes	–	–	–
	Rosen et al., 2020	Yes	Yes	–	–
	Weijer and Hart, 1983	Yes	–	–	No
	Weijer et al., 1972	Yes	–	–	–
Mast cell tumor, cutaneous	Dobromylskyj et al., 2015	Yes	–	–	–
	Johnson et al., 2002	–	–	–	Yes
	Lepri et al., 2003	–	Yes	–	–
	Melville et al., 2015	Yes	–	–	–
	Molander-McCrary et al., 1998	No	–	–	–
	Sabattini and Bettini, 2019	Yes	–	–	–
	Sabattini and Bettini, 2010	Yes	–	–	–
	Sabattini et al., 2013	Yes	Yes	–	–
Mast cell tumor, intestinal	Sabattini et al., 2016	Yes	–	–	–
Malignant maxillary tumors	Liptak et al., 2021	Yes	Yes	–	–
Melanocytic tumors, non-ocular	Chamel et al., 2017	No	–	No	–
	Pittaway et al., 2019	Yes	–	–	–

Tumor type	Reference (author and year of publication)	Association suggested with			
		Survival	Progression	Metastasis	Recurrence
	Sabattini et al., 2018	Yes	–	–	–
	Reck and Kessler, 2021	IPD	IPD	–	–
Melanoma, iris	Wiggans et al., 2016	–	–	Yes	–
Merkel cell tumor	Sumi et al., 2018	IPD	IPD	IPD	–
Oral tumors	Saverino et al., 2021	IPD	–	IPD	–
Osteosarcoma	Dimopoulou et al., 2008	–	–	–	Yes
Pancreatic tumor	Linderman et al., 2013	No	No	–	–
Plasma cell tumor	Craig and Lieske, 2022	IPD	–	IPD	–
Progressive histiocytosis	Coste et al., 2019	No	–	–	–
Salivary gland tumor	Hammer et al., 2001	Inverse	–	–	–
Soft tissue sarcoma, (mostly) skin	Bostock and Dye, 1979	Yes	–	–	No
	Guidice et al., 2010	–	–	–	No
	Kamenica et al., 2008,	No	–	–	No
	Porcellato et al., 2017	Yes	–	–	Yes
	Guisado and Castro, 2022	–	–	Yes	Yes
	Dobromylskyj et al., 2021	Yes	–	–	–
Squamous cell carcinoma, oral	Yoshikawa et al., 2016	No	No	–	–
	Yoshikawa et al., 2012	No	–	–	–
Squamous cell carcinoma, cutaneous	Rodriguez Guisado et al., 2021	Yes	–	–	–

Yes, significant results (p ≤ 0,05); No, non-significant results (p > 0,05); -, not evaluated; IPD, individual patient data (statistical association of the MC with outcome or authors conclusion is not provided)

Risk of bias

Supplemental Table S7. Summary of the risk of bias (RoB) for each evaluated domain (D1-4) and overall RoB for all studies on the mitotic index (MI) in feline tumors.

Article	Risk of bias				
	D1: Study population	D2: Outcome assessment	D3: MI method	D4: Data analysis	Overall (D1-4)
Preziosi et al. [1]	◯	◯	⊖	◯	◯
Sarli et al. [2]	◯	◯	⊖	◯	◯
Seixas et al. [3]	◯	◯	◯	◯	◯

⊕, low RoB; ◯, moderate RoB; ⊖, high RoB

Prognostic value

Supplemental Table S8. Prognostic value of the mitotic index (MI) in feline mammary carcinoma.

Article	Number of cases	Follow-up period	Tumor-specific survival time			
			Cut-off value [b]	MST, low MI value	MST, high MI value	Log rank test
Preziosi et al. [1]	33 [a]	2 years	0.719 % [b]	22.43 ± 88.78 months	12.37 ± 7.49 months	$p < 0.001$
Sarli et al. [2]	33 [a]	2 years	0.72 % [b]	N/A	N/A	$p < 0.001$
Seixas et al. [3]	64	2 years	1.1 % [b]	13 months	7 months	$p = 0.017$

MST, mean survival time; N/A, not available

[a] These two studies are presumed to have used the same study population

[b] the median of the MI values in the study population was used as the cut-off value

References

1. Preziosi R, Sarli G, Benazzi C, rioli L, Marcato PS. Multiparametric survival analysis of histological stage and proliferative activity in feline mammary carcinomas. *Research in Veterinary Science*. 2002;73**:** 53-60.
2. Sarli G, Preziosi R, Benazzi C, Bazzo R, rioli L, Marcato PS. Rate of apoptosis in feline mammary tumors is not predictive of postsurgical survival. *Journal of Veterinary Diagnostic Investigation*. 2003;15**:** 115-122.
3. Seixas F, Palmeira C, Pires MA, Bento MJ, Lopes C. Grade is an independent prognostic factor for feline mammary carcinomas: A clinicopathological and survival analysis. *Veterinary Journal*. 2011;187**:** 65-71.
